# Modified recombinant human erythropoietin with potentially reduced immunogenicity

**DOI:** 10.1038/s41598-020-80402-1

**Published:** 2021-01-15

**Authors:** Thanutsorn Susantad, Mayuree Fuangthong, Kannan Tharakaraman, Phanthakarn Tit-oon, Mathuros Ruchirawat, Ram Sasisekharan

**Affiliations:** 1 Program in Environmental Toxicology, Chulabhorn Graduate Institute, Bangkok, 10210 Thailand; 2 Translational Research Unit, Chulabhorn Research Institute, Bangkok, 10210 Thailand; 3 Koch Institute for Integrative Cancer Research, Massachusetts Institute of Technology, Cambridge, MA 02139 USA; 4 Department of Biological Engineering, Massachusetts Institute of Technology, Cambridge, MA 02139 USA

**Keywords:** Molecular modelling, Biologics

## Abstract

Recombinant human erythropoietin (rHuEPO) is a biopharmaceutical drug given to patients who have a low hemoglobin related to chronic kidney disease, cancer or anemia. However, some patients repeatedly receiving rHuEPO develop anti-rHuEPO neutralizing antibodies leading to the development of pure red cell aplasia (PRCA). The immunogenic antibody response activated by rHuEPO is believed to be triggered by T-cells recognizing EPO epitopes bound to MHC molecules displayed on the cell surface of APCs. Previous studies have reported an association between the development of anti-rHuEpo-associated PRCA and the HLA-DRB1*09 gene, which is reported to be entrenched in the Thai population. In this study, we used computational design to screen for immunogenic hotspots recognized by HLA-DRB1*09, and predicted seventeen mutants having anywhere between one through four mutations that reduce affinity for the allele, without disrupting the structural integrity and bioactivity. Five out of seventeen mutants were less immunogenic in vitro while retaining similar or slightly reduced bioactivity than rHuEPO. These engineered proteins could be the potential candidates to treat patients who are rHuEpo-dependent and express the HLA-DRB1*09 allele.

## Introduction

Erythropoietin (EPO) is a protein hormone produced by the kidneys and plays an essential role in the production and maturation of red blood cells (RBCs), which carry oxygen from the lungs to the rest of the body. EPO contains 165 amino acids, which contributes to the relative molecular mass of around 30,600 daltons. Post-translational modification of this protein results in the addition of 4 carbohydrate chains: 3 N-linked and 1 O-linked glycosylation^1–3^, following which the molecular weight of erythropoietin is roughly 40% increased from its original mass. Recombinant human erythropoietin (rHuEPO) is administered to patients who have lower hemoglobin levels because of their inability to produce enough endogenous erythropoietin. These include patients having chronic kidney disease (CKD), patients dependent on dialysis, HIV infection and malignancy. rHuEPO has also been used to accelerate erythropoiesis in surgery, post-chemotherapy and post-transplantation^4^. Indeed, treatment of anemia with rHuEPO has been shown to improve the quality of life (QoL) of these patients^5,6^.

Several reports have shown that the number of reported cases with PRCA due to the development of neutralizing antibodies against endogenous EPO and recombinant erythropoiesis-stimulating agents (ESAs) has increased worldwide^7–13^. Most of these reported cases were in patients receiving rHuEPO for the treatment of CKD-related anemia. The anti-rHuEPO neutralizing antibodies cross-react with endogenous EPO. The development of autoantibodies against endogenous EPO in patients who have never been treated with ESAs is very rare^14^. While PRCA is a type of anemia associated with several causes including virus infection, immunological mediation, pregnancy, pre-stage malignancy, toxic exposure, and drug effects, the distinct features of PRCA associated with rHuEPO includes severe rHuEPO resistance, blood transfusion dependence, high serum ferritin, bone marrow showing the absence of red cell precursor and presence of anti-rHuEPO antibody^15,16^. PRCA symptoms include very low reticulocyte and erythroid progenitor cells while other blood cell parameters are normal^15^. The incidence of EPO-associated PRCA is a significant burden on the affected population worldwide, especially in the most affected countries such as Thailand^17^.

The immunogenic antibody response activated by rHuEPO is believed to be T-cell dependent^16,18^. An antigen-presenting cell (APC) such as dendritic cell (DC) uptakes, processes and presents antigen as a peptide epitope to naïve T-cells using major histocompatibility complex (MHC) class II molecules on its surface. The binding between a MHC class II-epitope complex and T-cell receptor (TCR) fully activates the T-cell to release cytokines for the activation and differentiation of B cell to plasma cell. Plasma cell then secretes antibody against the corresponding epitope.

A study on the distribution of HLA alleles including A, B, DR and DQ among Thai patients having a proven anti-rHuEpo associated PRCA, CKD patients waiting for kidney transplant and normal people who donated the stem cells, demonstrated a correlation between the HLA-DRB1*09 and the anti-rHuEpo PRCA cases^17^. Additionally, this study demonstrated that all PRCA cases with HLA-DRB1*09 also showed HLA-DQB1*03:09^17^. The HLA-DRB1*09 allele is common in Thailand and some other regions such as China, Taiwan, Malaysia, Japan, Hong Kong, Samoa, Russia but it is rare in North America and Europe^19^. Hypersensitivity to rHuEPO might be higher in particular population. Several approaches have been developed to modulate the immunogenicity of EPO proteins^20,21^. Improvement of solution properties of biopharmaceuticals may also reduce immunogenicity because aggregates have been observed to be more immunogenic than soluble proteins^20^. Mutagenesis targeted at the HLA class II allele sites on EPO protein sequence that is most responsible for stimulating the immune system have been shown to lower immunogenicity in vitro. The engineered analog epitopes of rHuEPO were shown to have reduced HLA binding affinity. Two of the engineered mutations were proven to reduce in vitro immunogenicity^21^. Akin to this, this study aims to use computational methods to reduce immunogenicity of rHuEPO by altering the HLA immunodominant epitopes that are recognized by HLA-DRB1*09. The engineered rHuEPO could be potential alternate candidates for patients in Thailand and other regions who have immunogenicity to rHuEPO caused by high frequency of the HLA-DRB1*09 gene.

## Results

### Designing rHuEPO with lower immunogenicity in patients with HLA-DRB1*09 gene

**Figure 1:**
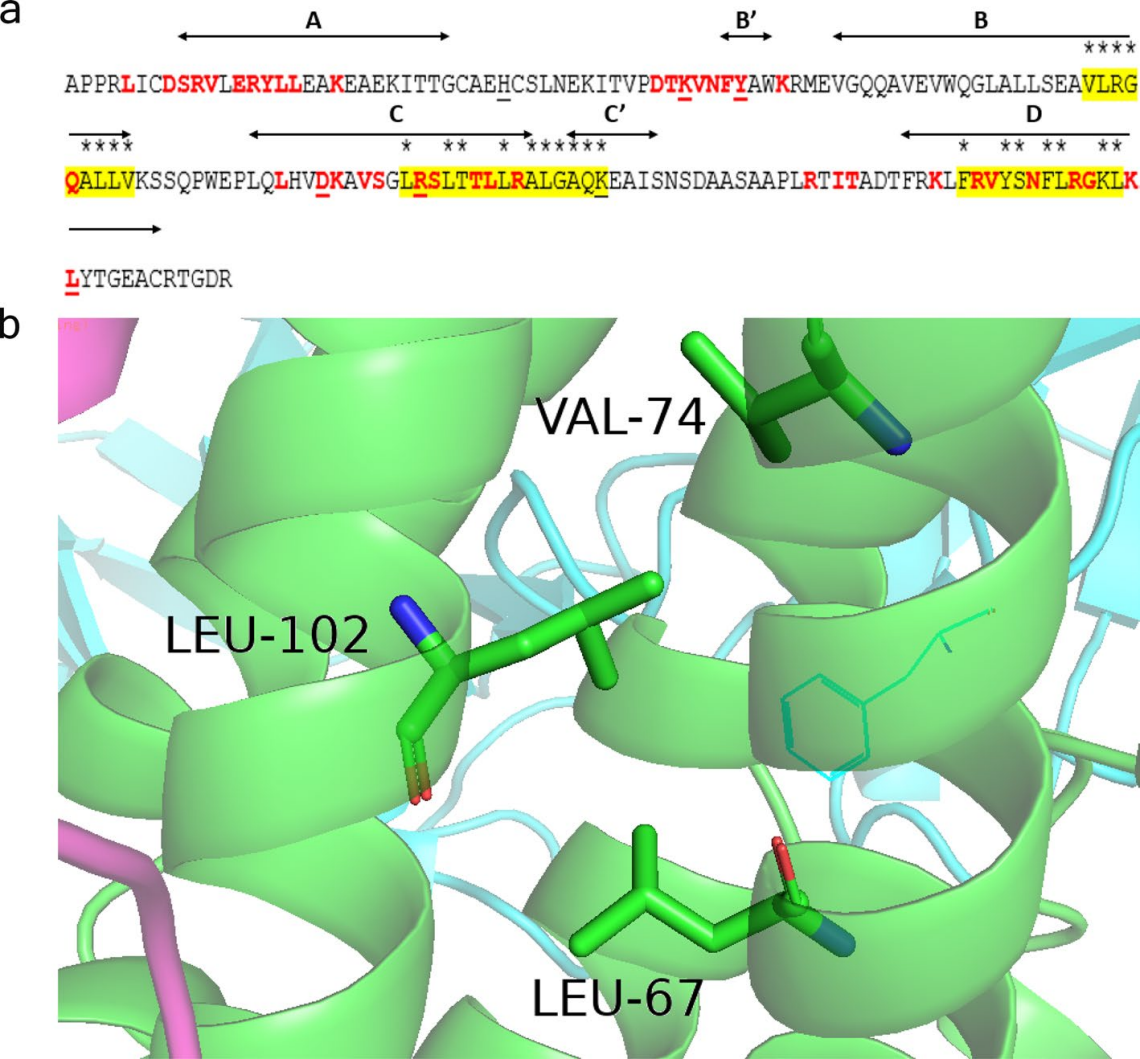
MHC epitope prediction analysis. (**a**) Shown is the amino acid sequence of EPO (residues 1–166). Sites contacted by the receptor (PDB: 1EER) are marked in red. Regions predicted by NetMHCII 2.2—a tool for HLA class II allele peptide prediction—for binding to HLA-DRB1*09-DQB1*03:09 to allele are highlighted in yellow. Sites recognized by neutralizing antibodies are underlined. Sites considered for amino acid modification are marked with an asterisk above. (**b**) Example of a network of interacting hydrophobic amino acids including two of allele binding sites, 74 and 102. Modification to 74 and/or 102 was then compensated with a suitable substitution at other positions in this network. The figure was generated using PyMOL version 2.3.4 (https://pymol.org/2/).

We carried out a computational assessment of MHC II (HLA-DRB1*09-DQB1*03:09) allele binding hotspots on EPO amino acid sequence with the idea of introducing mutations that would disrupt the binding with the HLA class II allele while not affecting the structure and binding of EPO receptor. We employed NetMHCII 2.2—a tool for HLA class II allele peptide prediction—to predict sites on EPO that would favor binding to HLA-DRB1*09-DQB1*03:09 allele^22^. The five core binding sites were predicted based on HLA-DRB1*09 (*09:01, *09:02, *09:03, *09:04, *09:05, *09:06, *09:07, *09:08, *09:09) to be VLRGQALLV (position 74–82), LRSLTTLLR (position 102–110), LLRALGAQK (position 108–116), FRVYSNFLR (position 142–150), and YSNFLRGKL (position 145–153), and this mapped to 3 distinct non-overlapping regions (residues 74–82 (VLRGQALLV), 102–116 (LRSLTTLLRALGAQK) and 142–153 (FRVYSNFLRGKL)) (Fig. 1a). However, no significant hits were found for HLA-DQB1*03:09. Interestingly, the predicted alleles overlapping with the sites recognized by the two cell-surface erythropoietin receptors, inferred from the co-crystal structure (PDB: 1EER). Known sites of neutralizing anti-EPO antibodies from the literature were also mapped. Our intention was to modify residues that are in the predicted allele binding regions but not involved in the EPO-R binding interface (Fig. 1b). We chose to focus more on regions 102–116 and 142–153 since they overlap or in proximity to known neutralizing antibody sites. This involved a total of 25 sites: 74, 75, 76, 77, 79, 80, 81, 82, 102, 105, 106, 109, 111, 112, 113, 114, 115, 116, 142, 145, 146, 148, 149, 152 and 153 (Fig. 1a). The design process had to factor the impact any amino acid mutation may have on the structure as allele sites considered for modification (Fig. 1b) were located within the highly networked helices (B-C-D) of the alpha-helical bundle. Because many of the sites were buried within the core of the four helical bundle structures, we engineered networks of interacting mutations, in many cases, to preserve structural integrity while disrupting allele binding (Fig. 1). Optimization of interatomic contacts was made using a variant of the EPCN method^23^. This exercise led to a total of 17 HLA-affinity-disrupting designs on EPO (Table 1).

**Table 1:** List of EPO mutations.

Name	Position(s) of mutation
EPO-WT	–
EPO-1.1	L67V, L102V
EPO-1.2	L70V, V74L, L102I
EPO-1.3	L102T
EPO-1.4	L67N, V74N, L102D
EPO-1.5	L67D, V74D, L102N
EPO-2.1	L12A, Y15I, L105F, L149W
EPO-2.2	L12I, Y15W, L105S, L149V
EPO-2.3	L105F
EPO-3.1	T106A
EPO-3.2	T106G
EPO-3.3	T106H
EPO-4.1	L109A
EPO-4.2	L109W
EPO-4.3	L109C, A114C
EPO-5.1	L102T, L105S, T106H, L109W
EPO-5.2	L102T, L105S, T106G, L109W
EPO-6.1	S146N, K152Q

### Characterization of the rHuEPO proteins

**Figure 2:**
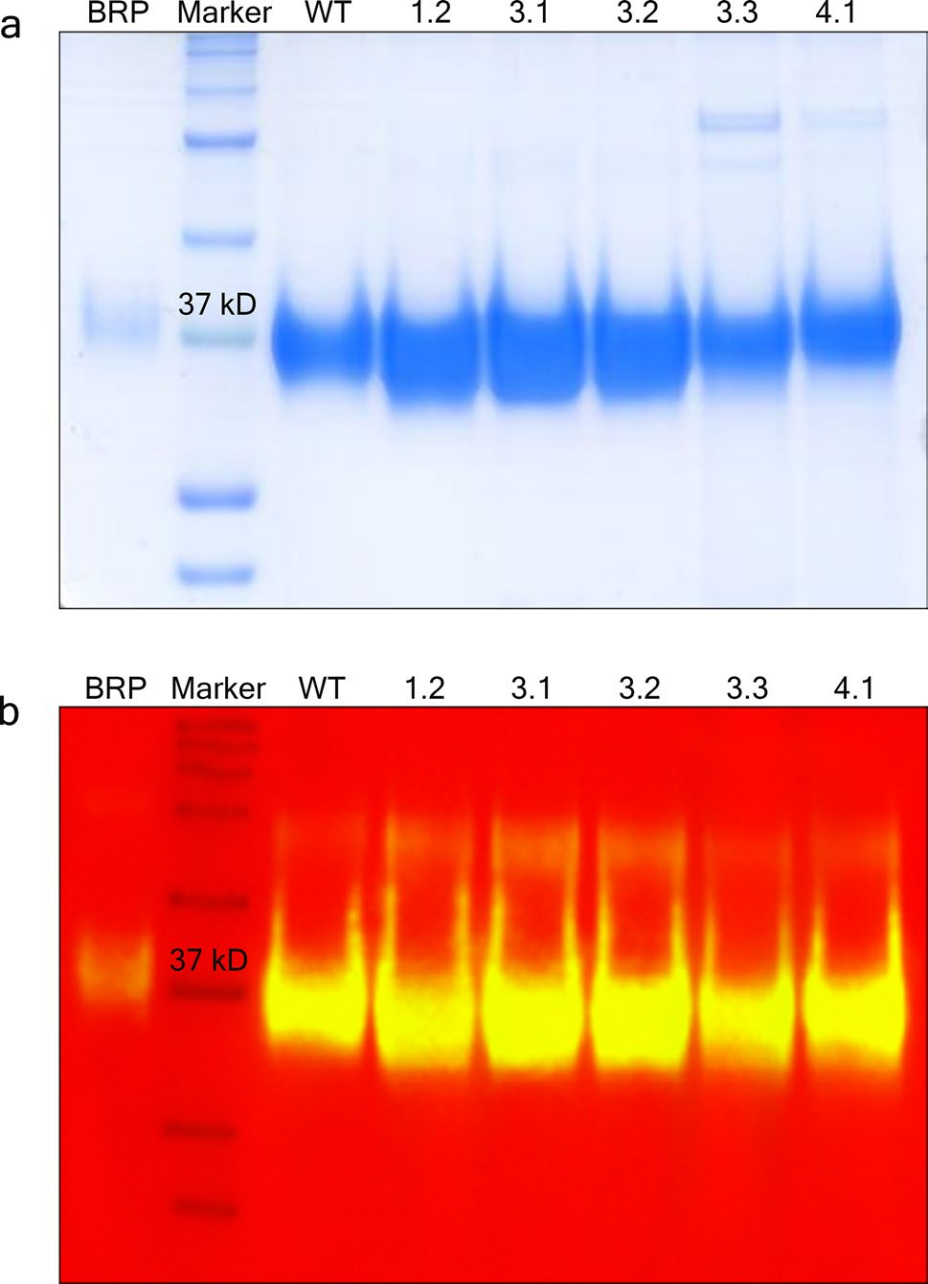
Coomassie blue stained gel (**a**) and Western immunoblotting (**b**) of the EPO proteins. EPO standard, wild type and mutant proteins were separated according to their molecular mass in 12% SDS polyacrylamide gel at a constant 175 voltages for 1.5 h. (**a**) The purity of separated proteins was visualized by Coomassie blue staining. The gel image was taken using HP LaserJet scanner. (**b**) The identity of EPO antigen was detected by Western immunoblotting. The chemiluminescence signals of protein samples were detected and the image was taken using ImageQuant LAS 4000 control software version 1.2 provided with ImageQuant LAS 4000 machine at the exposure time of 1 s. The signals were then overlaid with the marker using ImageQuant TL software version 7.0 (https://www.cytivalifesciences.com/en/us). The native gel and blot are presented in Supplementary Figs. S1 and S2, respectively. BRP, EPO standard; Marker, Precision Plus Protein Kaleidoscope Prestained Protein Standards; WT, EPO-WT; 1.2, EPO-1.2 (L70V, V74L, L102I); 3.1, EPO-3.1 (T106A); 3.2, EPO-3.2 (T106G); 3.3, EPO-3.3 (T106H); 4.1, EPO-4.1 (L109A).

Seventeen HLA-affinity-disrupting mutants of EPO were expressed in FreeStyle 293-F cell line and the proteins were purified and quantitated as described in the Materials and Methods. SDS-PAGE was used together with Coomassie blue staining to reveal the protein content in the purified EPO protein from FPLC (Fig. 2a). The size of deglycosylated and glycosylated EPO protein is estimated to be 30.6 kDa and 36–40 kDa, respectively^1^. The biological reference preparation (BRP) of EPO batch 4 (European Directorate for the Quality of Medicines & Health Care European Pharmacopoeia, France) was used as reference^24^. Both BRP, purified EPO-WT and EPO mutants showed expected molecular weight (Fig. 2a). Western blot analysis using anti EPO antibody showed the positive band for all samples including BRP, EPO-WT and EPO mutant proteins (Fig. 2b), confirming the identity of the proteins.

**Figure 3:**
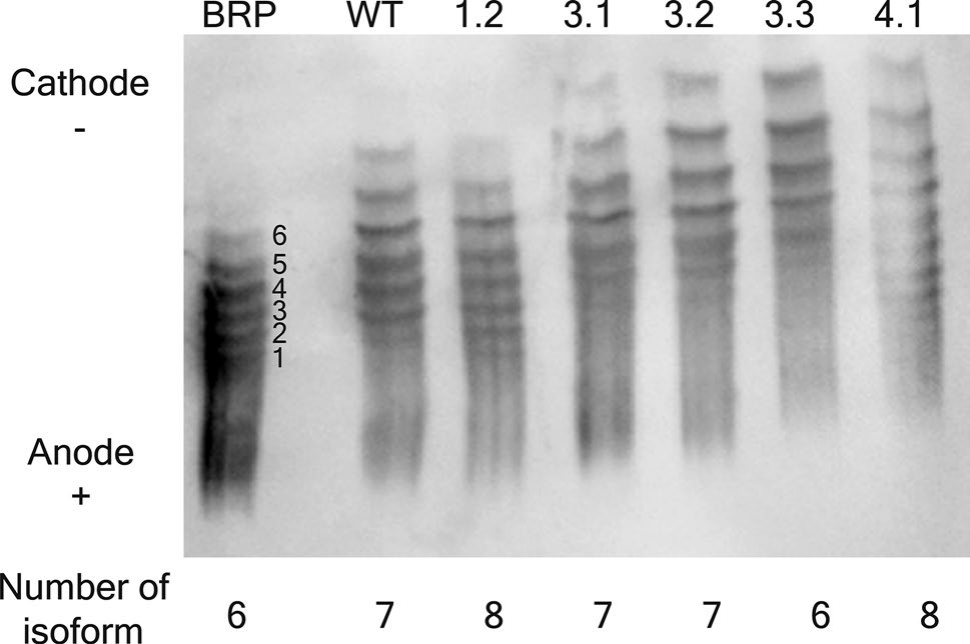
Isoform analysis by IEF of EPO proteins. EPO standard, wild type and mutant proteins were separated according to their pI in IEF gel with a pH gradient of 2–6 followed by Western blot. The chemiluminescence signals of protein samples were detected and the image was taken using ImageQuant LAS 4000 control software version 1.2 provided with ImageQuant LAS 4000 machine (https://www.cytivalifesciences.com/en/us) at the exposure time of 5 s. The native blot with multiple exposures is presented in Supplementary Fig. S3. The separated bands of the proteins represent the number of their existing glycoforms. Distribution of different isoforms of the purified EPO protein was compared to BRP, an EPO standard. BRP, EPO standard; WT, EPO-WT; 1.2, EPO-1.2 (L70V, V74L, L102I); 3.1, EPO-3.1 (T106A); 3.2, EPO-3.2 (T106G); 3.3, EPO-3.3 (T106H); 4.1, EPO-4.1 (L109A).

The carbohydrate portions of EPO contain sialic acid molecules. The variability in sugar structure and the number of sialic acid molecules also affect on EPO protein that exists as a mixture of isoforms. The sialic acid residues are necessary for biological activity including receptor binding affinity and serum half-life. Removal of sialic acid from EPO showed an increased in vitro bioactivity but reduced in vivo bioactivity^25^. The isoform distribution of the products was represented by the number of bands separated on the IEF gels. Purified EPO samples were separated on an IEF gel according to their pI. The glycoform distribution among BRP and 6 EPO samples (1 wild type and 5 mutants) were observed in the basic area of the gel (Fig. 3). According to an established method (TD2014EPO) for detection and reporting of rHuEPO using an electrophoretic technique provided by WADA, BRP showed 6 isoforms as expected. However, the number of isoforms among EPO samples varied (EPO-3.3 also showed 6 isoforms, EPO-WT, EPO-3.1 and EPO-3.2 showed 7 isoforms while EPO-1.2 and EPO-4.1 showed 8 isoforms). EPO-3.1, EPO-3.2, EPO-3.3 and EPO-4.1 showed the shift of the isoform distribution toward the basic isoforms. The two most basic bands of those EPO samples were significantly higher than the EPO-WT. While EPO-1.2 showed comparable profile or a slight shift toward the acidic isoforms. The isoform distribution was varied. This might be from the differences in the microheterogeneity of glycosylation between EPO samples. However, the higher isoforms of the proteins might exhibit an increase in bioactivity due to a decrease in serum clearance, which could be administered less frequently than the original drug.

### EPO mutations at the HLA-DRB1*09 core binding sites that retain its bioactivity

**Figure 4:**
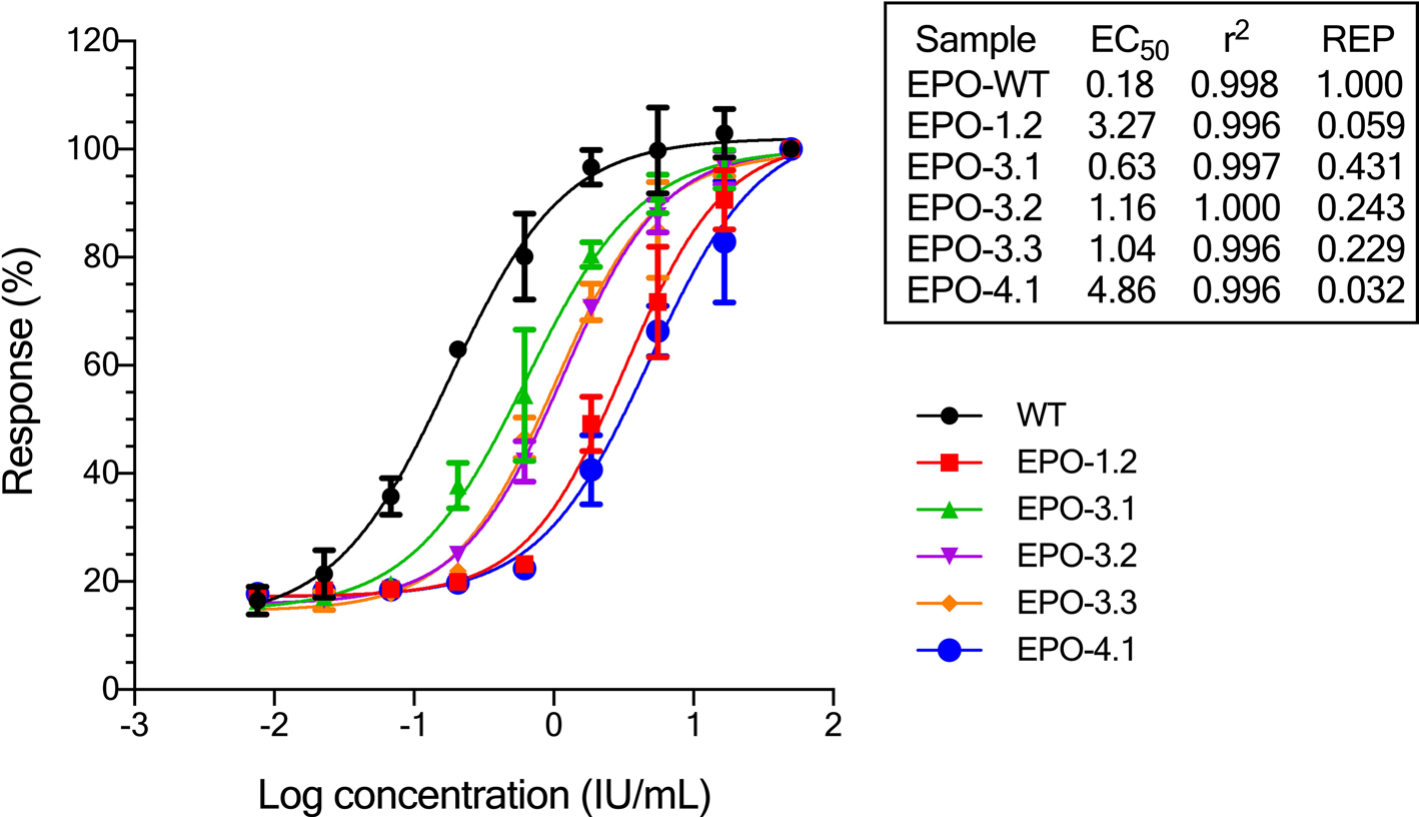
In vitro bioactivity of EPO-wild type and EPO mutants. EPO-WT ( ) and EPO mutants including EPO-1.2 ( ), EPO-3.1 ( ), EPO-3.2 ( ), EPO-3.3 ( ) and EPO-4.1 ( ) were tested for their bioactivity on TF-1 cells. Sample dilutions were done in quadruplicate. The absorbance reading corresponded to an increased cell number. The EC_50_, REP (relative activity compared to EPO-WT) and R^2^ of a 4-parameter logistic regression model were calculated using Gen5 data analysis software. The log dose–response curve of a 4-parameter logistic regression was generated by GraphPad Prism version 8.4.3 for Windows (https://www.graphpad.com/). WT, EPO-WT; 1.2, EPO-1.2 (L70V, V74L, L102I); 3.1, EPO-3.1 (T106A); 3.2, EPO-3.2 (T106G); 3.3, EPO-3.3 (T106H); 4.1, EPO-4.1 (L109A).

The bioactivity of mutated EPO proteins (Table 1) was measured in a cell proliferation assay using the TF-1 cell line. The TF-1 cell line, which expresses endogenous EPO receptors, has been derived from a patient with erythroleukemia. EPO also plays a role in the short-term growth of these cells and induces their differentiation into mature RBCs. In vitro bioactivity screening of EPO-WT and 17 mutants using TF-1 proliferation assay indicated that only 5 mutants showed bioactivity (EPO-1.2 (L70V, V74L and L1023I), EPO-3.1 (T106A), EPO-3.2 (T106G), EPO-3.3 (T106H), and EPO-4.1 (L109A)) (Fig. 4). All 5 mutants showed a lower bioactivity than the wild-type, especially EPO-1.2 and EPO-4.1 with the relative bioactivity (REP) to EPO-WT of 0.059 (or 5.9% activity compared to EPO-WT) and 0.032 (or 3.2% activity compared to EPO-WT), respectively. EPO-3.1 had a highest bioactivity among EPO mutants with REP of 0.431 (or 43.1% activity compared to EPO-WT). EPO-3.2 and EPO-3.3 were in the same range of bioactivity with REP of 0.243 (or 24.3% activity compared to EPO-WT) and 0.229 (or 22.9% activity compared to EPO-WT), respectively.

### Immunogenicity test of purified rHuEPO proteins

**Figure 5:**
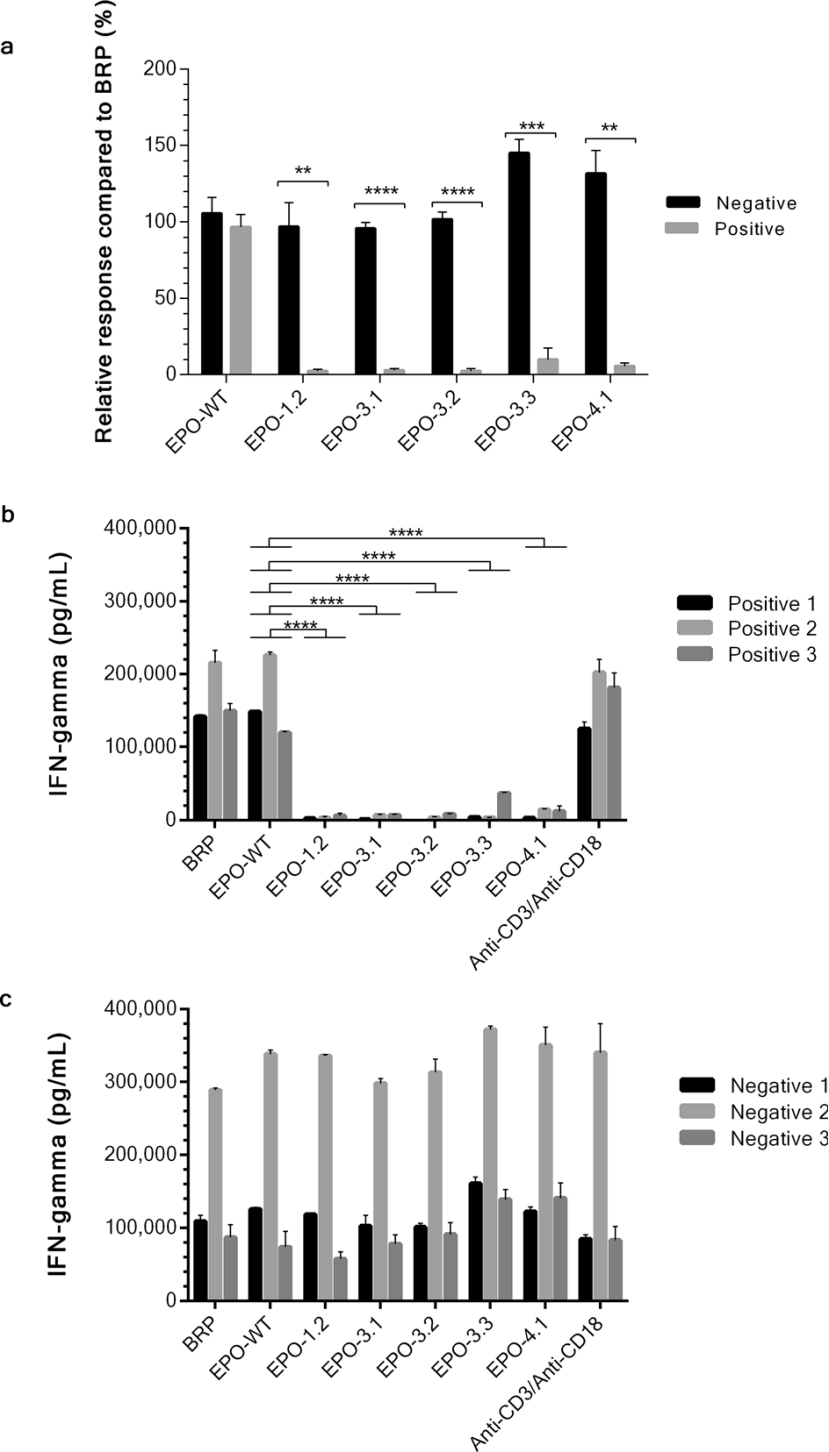
Ex vivo immunogenicity test of EPO proteins. Mature DC cells were pulsed with EPO protein including EPO-1.2 (L70V, V74L, L102I), EPO-3.1 (T106A), EPO-3.2 (T106G), EPO-3.3 (T106H) or EPO-4.1 (L109A). Anti-CD3/anti-CD28, the known CD4^+^ T cell activator was used as a positive control. EPO-pulsed DCs and CD4^+^ T cell from the same individual were cocultured at a 1:20 ratio. CD4^+^ T cell activation was quantified by the measurement of IFN-γ release using sandwich ELISA. The mean value from 3 HLA-DRB1*09-negative volunteers and 3 HLA-DRB1*09-positive volunteers were combined as a negative group or a positive group, respectively. Each group was relatively compared to the response to BRP and presented in the percentage of relative response of EPO proteins shown in **(a)**. Bar graphs are shown as mean ± SEM calculated from 3 individuals in each group. Asterisks denote a significant difference by *t*-test (* *p* ≤ 0.05, ** *p* ≤ 0.01, *** *p* ≤ 0.001, **** *p* ≤ 0.0001). IFN-γ level of each HLA-DRB1*09-positive volunteers shown in **(b)** and HLA-DRB1*09-negative volunteers shown in **(c)**. Bar graphs are shown as mean ± SEM calculated from duplicate experiments. The similarity or difference (**** *p* ≤ 0.0001) in IFN-gamma level among BPR, EPO-WT, EPO mutants and anti-CD3/anti-CD28 in both positive and negative group was analyzed by one-way ANOVA followed by Tukey’s multiple comparisons test for (**b**) and (**c**). The significant differences were only shown in comparison to EPO-WT in this figure. Graphs were generated using GraphPad Prism version 8.4.3 for Windows (https://www.graphpad.com/).

Next, the EPO mutants that retain bioactivity were tested for their ability to reduce immunogenicity using an ex vivo assay. Blood samples from 50 healthy volunteers were collected and expression of HLA-DRB1*09 was determined since the mutations were designed based on HLA-DRB1*09 alleles. Eleven out of fifty volunteers (22%) had HLA-DRB1*09, of which there were four women (36%) and seven men (64%). All HLA-DRB1*09 negative and positive volunteers were randomly selected for immunogenicity test and were confirmed to be negative for common infectious diseases including hepatitis B, hepatitis C, syphilis, and HIV. To test whether the engineered EPO proteins with disrupted HLA-DRB1*09 binding affinity were less immunogenic, PBMCs from 3 HLA-DRB1*09 negative volunteers and 3 HLA-DRB1*09 positive volunteers were isolated. The high resolution of HLA-DRB1 types of these 6 volunteers are presented in Supplementary Table S1. Precursor DCs were separated from other immune cells by adherence to plastic whereas non-adherent cells were used as a source of CD4^+^ T cells. Maturation of precursor DCs to immature DCs by determination of HLA-DR expression was monitored by flow cytometry using FITC-conjugated anti-human HLA-DR. Percentage of immature DC maturation of both groups ranged from 50.7 to 80.4 on day 7 of DC culture. To generate mature DCs as an antigen presenting cells, immature DCs were pulsed with BRP or EPO protein (EPO-WT, EPO-1.2, EPO-3.1, EPO-3.2, EPO-3.3 and EPO-4.1) for 48 h. CD4^+^ T cells were isolated from the nonadherent cell fraction from the same volunteer who provided PBMC. Subsequently, EPO-pulsed DCs and CD4^+^ T cells were cocultured at a 1:20 ratio. Since anti-CD3 and anti-CD28 antibodies are known to stimulate T cells in a manner that partially mimics stimulation by APCs^26^. Therefore, the incubation with anti-CD3/anti-CD28 antibodies was used as a positive control, which activated T cell response. IFN-γ released by CD4^+^ T cells as a T cell response was quantified by sandwich ELISA. Level of IFN-γ from coculture between unpulsed DC and CD4^+^ T cells was considered as a background of T cell response. Statistical comparison of the relative T cell response of EPO protein compared to BRP between HLA-DRB1*09-negative and positive groups demonstrated that all 5 EPO mutants had a significant lower T cell response in HLA-DRB1*09-positive group. The *p* values were 0.00382 for EPO-1.2, 0.00002 for EPO-3.1, 0.00004 for EPO-3.2, 0.00031 for EPO-3.3 and 0.00116 for EPO-4.1.There was no difference in T cell response using EPO-WT between HLA-DRB1*09-negative and positive groups (Fig. 5a). The level of IFN-γ release from each HLA-DRB1*09-positive and negative volunteers is shown in Fig. 5b,c, respectively. In both HLA-DRB1*09-positive and negative group, BRP and EPO-WT could stimulate T cell response in the same manner as anti-CD3/anti-CD28 antibodies. There were no statistically significant differences among BRP, EPO-WT and anti-CD3/anti-CD28 antibodies in both positive and negative groups. In Fig. 5b, EPO mutants including EPO-1.2, EPO-3.1, EPO-3.2, EPO-3.3 and EPO-4.1 exhibited a lower T cell response with the IFN-γ release ranging from 220 to 37,000 pg/mL in positive group. As compared to EPO-WT, all EPO mutants showed the significant differences (*p* ≤ 0.0001). Figure 5c showed data from negative group; and EPO-WT and all EPO-mutants showed no significant differences in T cell response as compared to anti-CD3/anti-CD28 antibodies.

The different baseline of EPO-specific T cell response seen in each volunteer might be due to the difference in HLA allele distribution in individuals supported by 2 studies. Praditpornsilpa et al.^17^ demonstrated the high allele frequency of anti-rHuEpo associated PRCA cases and various HLA types including HLA-A (*02, *11 and *24), HLA-B (*18, *46, *60 and *62), HLA-DR (DRB1*09, DRB1*12, and DRB1*15). However, only HLA-DRB1*09 showed a strongest correlation with a statistical significance. Tangri et al.^21^ also observed different levels of T-cell response from five EPO-responsive PBMCs generated from donors having different HLA-DRB alleles for example HLA-DRB1*01:01, HLA-DRB1*03:01, HLA-DRB1*13:01, HLA-DRB3*01:01 and HLA-DRB4*01:01. These studies clearly illustrated the antigenicity of EPO among various HLA types. In conclusion, the engineered mutants exhibit lower immunogenicity while retaining key structural characteristics and bioactivity as summarize in Table 2.

**Table 2:** A summary of the 5 candidates EPO mutants, their relative effect on immunogenicity, bioactivity and predicted MHC binding.

EPO mutant	Core binding site(s)	Predicted MHC binding (nM)	Bioactivity relative to EPO-WT (%)	Immunogenicity with significant lower T cell response in HLA-DRB1*09-positive group
EPO-1.2	VLRGQALLV LRSLTTLLR	36.28 16.22	5.9	** p value = 0.00382
EPO-3.1	LRSLTTLLR	16.22	43.1	**** p value = 0.00002
EPO-3.2	LRSLTTLLR	16.22	24.3	**** p value = 0.00004
EPO-3.3	LRSLTTLLR	16.22	22.9	*** p value = 0.00031
EPO-4.1	LRSLTTLLR LLRALGAQK	16.22 14.2	3.2	** p value = 0.00116

### Predicted binding between EPO mutants and common HLA class II alleles

In order to assess the potential impact of the engineered mutations in a broader context, NetMHCIIpan version 3.2 was used to predict the peptide binding affinity to MHC class II molecules^22^. The 2 binding regions (15-mer peptides 68–82 and 104–118) within EPO-WT and the engineered mutants (EPO-1.2, EPO-3.1, EPO-3.2, EPO-3.3 and EPO-4.1) to the most common 15 DR, 6 DQ and 5 DP alleles that are prevalence in global population were scanned (Table 3)^27^. In particular, 7 DR alleles are common in Southeast Asia population including DRB1*07:01, DRB1*09:01, DRB1*11:01, DRB1*12:01, DRB1*15:01, DRB1*04:05, and DRB1*03:01^19^. The predicted affinity was shown in term of a percentile rank. A percentile rank for a peptide was generated by comparing its affinity against the scores of 200,000 random natural peptides of the same length of the query peptide. A weak binder was identified if the % rank was below 25%. A strong binder was identified if the % rank was below 2%. The fragments 68–82 and 104–118 of EPO-WT had varying affinities for the various alleles, with up to 115-fold variation. Except EPO-4.1, none of the designs showed greater than two-fold variation in binding compared to EPO-WT. EPO-4.1 showed two to five-fold decrease in affinity to 9/26 alleles (DRB1*01:01, DRB1*11:01, DRB1*12:01, DRB1*15:01, DRB1*04:01, DRB1*04:05, DRB4*01:01, DRB5*01:01, DPA1*02:01-DPB1*05:01) and three to six-fold increase in affinity to only 2/26 alleles (DQA1*05:01-DQB1*03:01 and DQA1*01:02-DQB1*06:02) (Table 3). Although, the peptides 68–82 and 104–118 of EPO-WT had appreciable affinities for DRB1*09:01 suggesting that these motifs are potential binding sites of the allele. The mutants however did not show a drop in binding, as expected, contrasting the findings of our ex vivo experiment. Collectively, the results of the in silico analysis show that (1) the engineered mutations do not pose any risk of increased immunogenicity due to the common alleles and (2) of all the designed variants, EPO-4.1 shows the highest potential to exhibit reduced immunogenicity relative to EPO-WT.

**Table 3:** Predicted binding between EPO variants and common HLA class II alleles.

Allele	Affinity to peptide 68–82 (% Rank)						Affinity to peptide 104–118 (% Rank)					
	EPO-WT	EPO-1.2	EPO-3.1	EPO-3.2	EPO-3.3	EPO-4.1	EPO-WT	EPO-1.2	EPO-3.1	EPO-3.2	EPO-3.3	EPO-4.1
**DR alleles**												
DRB1*01:01	10	6.5	10	10	10	10	4	4	3.5	3	2.5	8.5
DRB1*07:01	18	13	18	18	18	18	28	28	26	26	22	28
DRB1*09:01	13	9.5	13	13	13	13	15	15	14	13	12	13
DRB1*11:01	30	28	30	30	30	30	0.9	0.9	1.5	1.8	0.9	2.5
DRB1*12:01	11	8.5	11	11	11	11	5	5	4.5	6	3.5	25
DRB1*15:01	22	15	22	22	22	22	8	8	7.5	8	6.5	27
DRB1*04:01	26	36	26	26	26	26	13	13	12	11	10	27
DRB1*04:05	31	38	31	31	31	31	9	9	9	8	7.5	36
DRB1*08:02	19	26	19	19	19	19	1.3	1.3	1.7	2.5	1.1	1.9
DRB1*03:01	18	22	18	18	18	18	35	35	34	34	32	50
DRB1*13:02	12	11	12	12	12	12	44	44	43	42	42	60
DRB3*01:01	36	33	36	36	36	36	80	80	80	80	80	95
DRB3*02:02	40	31	40	40	40	40	30	30	29	28	27	40
DRB4*01:01	7.5	13	7.5	7.5	7.5	7.5	3.5	3.5	3	3	2.5	13
DRB5*01:01	29	28	29	29	29	29	0.7	0.7	0.6	0.5	0.6	1.5
**DQ alleles**												
DQA1*05:01-DQB1*02:01	35	38	35	35	35	35	31	31	30	32	32	42
DQA1*05:01-DQB1*03:01	18	23	18	18	18	18	25	25	23	22	25	7
DQA1*03:01-DQB1*03:02	34	37	34	34	34	34	19	19	18	19	19	14
DQA1*04:01-DQB1*04:02	30	33	30	30	30	30	12	12	12	12	13	6.5
DQA1*01:01-DQB1*05:01	35	30	35	35	35	35	44	44	41	44	42	75
DQA1*01:02-DQB1*06:02	6	8	6	6	6	6	4	4	4	4	5.5	0.6
**DP alleles**												
DPA1*02:01-DPB1*01:01	21	17	21	21	21	21	43	43	41	45	40	65
DPA1*01:03-DPB1*02:01	26	20	26	26	26	26	48	48	46	50	45	70
DPA1*01:03-DPB1*04:01	27	21	27	27	27	27	43	43	41	46	41	65
DPA1*01:03-DPB1*04:02	23	18	23	23	23	23	32	32	31	35	31	50
DPA1*02:01-DPB1*05:01	19	15	19	19	19	19	19	19	17	21	17	38

## Discussion

Since the rHuEPO become more available for treatment of anemia of patients having CKD, some CKD patients who have previously or are currently using rHuEPO have been reported to display suspected or confirmed PRCA^17^. A report in 2005 indicated that 4 Thai patients who received rHuEPO therapy had a confirmed anti-rHuEPO associated PRCA and all of them also displayed HLA-DRB1*09^17^. DRB1*09 allele has a high frequency among Southeast Asian countries. The immunogenic adverse effect of rHuEPO is proven to be T-cell dependent. The disruption between EPO epitope and HLA-DRB1*09 molecule is predicted to lower immunogenicity in patients with HLA-DRB1*09. We employed computational methods to modify EPO via rational protein engineering to have reduced binding to HLA-DRB1*09.

Both EPO-WT and EPO mutants were screened for their biological activity using in vitro TF-1 proliferation assay. This cell type exhibits EPO receptor on its surface and shows commitment to erythroid lineage and has an intact Jak2/Stat5 pathway, which mediates cell proliferation in response to various growth factors including EPO^28,29^. Five out of seventeen EPO mutants (EPO-1.2, EPO-3.1, EPO-3.2, EPO-3.3 and EPO-4.1) exhibited in vitro bioactivity as compared to wild type. However, the variation in bioactivity on TF-1 cells may due to the differences in protein sequences, overall protein conformation and glycosylation pattern. It is well known that rHuEPO has a complex glycosylation pattern and exists in a group of different isoforms. The isoform of rHuEPO play an important role in their activity, potency and also stability^25^. It has been shown that in vivo activity has greatly decreased in non-glycosylated EPO. Fully glycosylated EPO is known to contain as many as 14 isoforms. IEF result showed the correct glycoforms of BRP (6 different glycoforms) indicating that the test conditions were valid and other samples could be compared. The glycoform patterns of 6 EPO samples (EPO-WT, EPO-1.2, EPO-3.1, EPO-3.2, EPO-3.3 and EPO-4.1) were varied ranging from 6 to 8 isoforms. This variation of glycoform patterns may be due to the difference in protein sequence and posttranslational modifications of the glycan moieties by the different passage of transfected cells. However, a higher glycosylation increases persistence of EPO, resulting in a prolonged serum half-life^30–34^. The variation of isoform distribution and isoform abundance in each EPO proteins may also explain the variation of bioactivity among these proteins.

In terms of the structure and function relationship, EPO structure has a 4-helical bundle topology (helices A, B, C and D). The mutations involved in EPO-1.2, EPO-3.1, EPO-3.2, EPO-3.3 and EPO-4.1 that had in vitro bioactivity were located on helix B and C, which are important for the stability of its conformation. Sites 74, 102, 106 and 109 are found to be an epitope for HLA-DRB1. Alanine, leucine, valine and isoleucine are in the same group of non-polar aliphatic amino acids. They are interchangeable. Substitution of leucine to valine at position 70, valine to leucine at position 74, leucine to isoleucine at position 102 (EPO-1.2) and leucine to alanine at position 109 (EPO-4.1) are considered to preserve the hydrophobic network of wild type EPO. On the other hand, the single mutants involved in EPO-3.1, EPO-3.2 and EPO-3.3 alter the native amino acid character. Polar hydroxyl-containing amino acid threonine at position 106 was changed to smaller and non-polar amino acids, which are alanine (EPO-3.1) and glycine (EPO-3.2). EPO-3.3, on the other hand, was modified to have an imidazole side chain at 106 to avoid recognition of HLA class II allele. All EPO candidates containing these mutated sequences might be considered as the altered HLA epitope that might not be recognized by the HLA molecule leading to the lower immunogenic response in patient who uses EPO and has this HLA.

To test this hypothesis, the immunogenicity of EPO protein was evaluated using PBMC from HLA-DRB1*09-negative and positive volunteers. The screening of HLA-DRB1*09 in volunteers living in Bangkok, Thailand found that 22% volunteers have HLA-DRB1*09 confirming the high frequency of this particular HLA in Thailand. Because the types of MHC molecules expressed in laboratory animal are different from human, therefore, we used an ex vivo human primary model rather than animal model. Due to the small study group, apart from *t-test*, we also confirmed the statistically significant differences of the T cell response among EPO mutants between HLA-DRB1*09-negative and positive groups using the non-parametric Mann–Whitney U test. The statistical differences at *p* ≤ 0.01 were obtained for all EPO mutants compared to the EPO-WT (data not shown). The statistical differences at *p* ≤ 0.01 were obtained for all EPO mutants compared to the EPO-WT (data not shown). The magnitude of immunogenicity observed in all 5 EPO mutants was significantly decreased in HLA-DRB1*09-positive volunteers when compared to HLA-DRB1*09-negative volunteers. These altered proteins with reduced immunogenicity could associate with the reduced HLA-DRB1*09 binding affinity. A study by Tangri et al. identified EPO epitopes and modified those epitopes in EPO protein to reduce HLA-DR binding. The modified EPO protein showed bioactivity and lower immunogenicity^21^. The mutations reported in this paper are distinct from Tangri et. al.

The potential impact of the engineered mutations in a broader context was assessed and indicated that the engineered mutations do not pose any risk of increased immunogenicity due to the common alleles in Southeast Asia and global populations. However, the in silico prediction did not show the drop in binding affinities of the engineered mutations to DRB1*09:01 allele as compared to the ex vivo data in this study. This apparent contradiction could be due to limitations in capturing the effects of site-specific modifications on allele binding. We noted that the design of the EPO variants did not rely on comparing the score pre- and post-modification. Rather the design process was governed by several sequence and structural factors such as: (1) the location of the site to be modified within the core peptide, (2) proximity to known neutralizing antibody sites (3) whether the modification will have any impact on binding EPO receptor, and (4) whether the modification will destabilize the structure by affecting interatomic networks.

Due to a high incidence of rHuEPO-associated PRCA cases and high frequency of HLA-DRB1*09 in Thailand, here, we successfully used in silico tool to design EPO mutants to disrupt the EPO-T cell interaction. The mutation was made to reduce the binding between EPO epitope and HLA-DRB1*09 paratope. Five out of seventeen mutants exhibited biopotency confirming their unaffected biological function. A significant decrease in T cell response in HLA-DRB1*09-positive volunteers demonstrated that these engineered proteins could be the potential alternative candidates for patients who have HLA-DRB1*09 gene with no potential impact on the increased immunogenicity to the other common alleles found in Southeast Asia and global populations.

## Materials and methods

### Cell culture

FreeStyle 293-F cell (Invitrogen) were cultivated at 37 °C under 8% CO_2_ in FreeStyle 293 Expression Medium (Invitrogen) using a shaker at 130 RPM. Human erythroleukemic cell line, TF-1 (ATCC) were cultivated at 37 °C under 5% CO_2_ in RPMI 1640 medium (Thermo Fisher Scientific) supplemented with 10% FBS, 1% Pen-strep and 2 ng/mL GM-CSF.

### MHC epitope prediction analysis

In silico tool was used to predict amino acid residues responsible for binding between EPO epitopes and HLA class II allele HLA-DRB1*09-DQB1*03:09. The residues responsible for maintaining the structural integrity of the EPO protein and EPO receptor binding were unchanged in order to maintain its bioactivity. The non-receptor-binding site (non-RBS) residues were modified to reduce the HLA and neutralizing antibody binding. The interatomic contacts were optimized using a variant of the EPCN method^23^. Seventeen EPO mutants were designed (Table 1).

### Affinity prediction to common HLA class II alleles

The binding prediction of EPO-WT and mutants (EPO-1.2, EPO-3.1, EPO-3.2, EPO-3.3 and EPO-4.1) to the common HLA class II alleles was performed using NetMHCIIpan version 3.2 (http://www.cbs.dtu.dk/services/NetMHCIIpan-3.2/). The two regions (15-mer peptides 68–82 and 104–118) were used for a peptide scan. The common 15 DR alleles, 6 DQ alleles and 5 DP alleles were included in the analysis. NetMHCIIpan version 3.2 was run with the following settings: peptide length to scan: 15, threshold for weak binder (% rank) < 25%; threshold for strong binder (% rank) < 2%, where the percentile rank for a peptide is generated by comparing its affinity against the scores of 200,000 random natural peptides of the same length of the query peptide.

### Plasmid generation and preparation

A pcDNA 3.3 expression vector containing a sequence encoding EPO mutant having only 1 mutation/substitution site was generated using QuikChange II XL Site-Directed Mutagenesis Kit (Agilent Technologies). A sequence encoding for EPO mutant having more than 1 mutation/substitution sites was generated using gene synthesis, cloned into pcDNA 3.3 expression vector, and transformed into *E. coli* (DNA 2.0). Purified plasmids were submitted for DNA sequencing (Genewiz) to confirm the mutations.

### EPO protein expression and purification

The purified pcDNA 3.3 expression vector containing a sequence encoding EPO-WT or EPO mutant was transiently transfected into FreeStyle 293-F cell using 25 kD linear polyethylenimine (Polysciences). After 6 days, EPO protein was purified from culture supernatant using Hitrap Blue HP column (GE Healthcare), eluted with 1.5 M NaCl, and buffer exchanged into 20 mM Tris-HCI pH 8.45. Next, sample was loaded onto Hitrap Q HP column (GE Healthcare). The column was washed with 20 mM sodium acetate pH 4.00 followed by second wash with 20 mM Tris. EPO protein was then eluted with 1 M NaCl. Purified EPO protein was buffer exchanged into a storage buffer (50 mM sodium phosphate buffer, pH 7.0, 1.5% glycine and 0.003% tween-20).

### Quantification of EPO protein using sandwich ELISA

Sandwich ELISA was developed for quantitation of both EPO wild type and mutant proteins. A pre-absorbed Maxisorp 96-well plates (Nunc) was coated with 2.5 μg/mL of capture antibody (mouse monoclonal IgG_2A_ to human EPO, MAB 2871) (R&D Systems) in phosphate-buffered saline (PBS) and incubated at 4 °C overnight. Plate was washed three times with PBS. A blocking solution of 1% BSA in PBS plus 0.05% tween (PBST) was added. The plate was incubated at room temperature for 1 h and washed with PBST. The biological reference preparation (BRP) of EPO batch 4 (European Directorate for the Quality of Medicines & Health Care European Pharmacopoeia, France)^24^ was used to generate a standard curve. Serial dilution (2.5-fold) of the EPO standard or EPO samples was done with the starting concentration of 10,000 International Unit (IU) or undiluted concentration, respectively. Dilutions were done in PBST and in duplicate. Both BRP and the samples were incubated at room temperature for 2 h and then washed with PBST. The primary antibody (rabbit polyclonal IgG to human EPO, AB-286-NA) (R&D Systems) at a concentration of 1 μg/mL in PBST was added. Plate was incubated at room temperature for 1 h and washed with PBST. Subsequently, 0.1 μg/mL of secondary antibody (goat polyclonal secondary Ab to Rabbit IgG- H&L (HRP), pre-absorbed, ab97080) (Abcam) in PBST was added. Plate was incubated at room temperature for 1 h and then washed with PBST. For detection, TMB substrate (SeraCare) was added. Quenching was done by adding 1 N sulfuric acid. Plate was read using a microplate reader (Biotek) at 450 nm.

### In vitro bioactivity test of EPO protein

A total of 2.5 × 10^4^ TF-1 cells were plated into a 96-well flat-bottom plate (Nunc). Fifty International Unit per milliliter of BRP or EPO protein was three-fold diluted. All dilutions were done in quadruplicate. After 72 h incubation, WST-1 Cell Proliferation Reagent (Roche) was added. The reaction was incubated in 5% CO_2_ for 4 h. The absorbance of 450 nm and 690 nm were measured using a microplate reader (Biotek). Gen5 data analysis software (Biotek) was used to calculate EC_50_ and R^2^ using a 4-parameter logistic regression function. Parallelism analysis was performed for evaluation of relative potency (REP) of EPO mutant against EPO-WT. The log dose–response curve of a 4-parameter logistic regression was generated using GraphPad Prism version 8.4.3 for Windows (Graphpad Software, Inc.).

### Volunteers and preparation of peripheral blood mononuclear cells (PBMCs)

This study was approved by the human research ethics committee, Chulabhorn Research Institute, Thailand. All methods involving human participants were performed in accordance with the guidelines and regulations of the institutional ethical committee. All volunteers gave a written informed consent. Blood was collected from healthy volunteers in EDTA‐treated vacuum tubes (Becton Dickinson). HLA-DRB1 type was determined using sequence-specific oligonucleotide primed PCR (PCR-SSO) with the use of the Luminex technology at Faculty of Medicine, Chulalongkorn University, Thailand. Common infectious diseases including hepatitis B, hepatitis C, syphilis and HIV were also screened. PBMCs were isolated using IsoPrep solution (Robbins Scientific Corporation).

### Dendritic cell (DC) culture and flow cytometric analysis

A total of 10 × 10^6^ cells of PBMCs in 3 mL complete RPMI medium were plated in each well of a 6-well plate. After incubation at 37 °C under 5% CO_2_ for 2 h, the nonadherent cells were collected as a source of CD4^+^ T cells. The complete RPMI medium containing 50 ng/mL GM-CSF and 50 ng/mL IL-4 (ImmunoTools) was added on the adherent cells representing the precursor DCs. Cells were incubated and 75% spent medium was exchanged to fresh medium with cytokines every second day. The differentiation of precursor DCs to immature DCs was monitored by a BD FACSCanto Flow cytometer (BD Biosciences) using FITC-conjugated anti-human HLA-DR (ImmunoTools).

### Generation of mature DCs

Immature DCs were harvested and washed with complete RPMI medium. A total of 10^5^ cells were plated in each well of a 24-well plate and incubated (pulsed) with 10 µg/mL BRP or EPO protein for 48 h. Immuture DCs incubated without protein were used as control DCs (unpulsed DCs).

### Separation and activation of CD4^+^ T cells

CD4^+^ T cells were prepared from the nonadherent cell fraction of PBMCs from the same volunteer who provided monocytes for the DC culture. CD4^+^ T cells were isolated by negative selection using MACS Human CD4^+^ T cell Isolation Kit (Miltenyi Biotec). Viability was greater than 90% (data not shown). To activate effector immune cells, EPO-pulsed (mature) DCs and CD4^+^ T cells derived from the same volunteer were cocultured at a 1:20 ratio. Positive control included the coculture of unpulsed DC and CD4^+^ T cells supplemented with 5 µg/mL anti-human CD3 and 2 µg/mL anti-human CD28 (ImmunoTools). Background control included the coculture of unpulsed DC and CD4^+^ T cells alone. All cocultures were incubated in the presence of 30 ng/mL IL-2 and 30 ng/mL IL-12 (ImmunoTools) for 4 days and then harvested. The level of IFN-γ representing T cell response was quantified by Human IFN gamma ELISA Kit (ImmunoTools).

### SDS-PAGE and Coomassie blue staining

Protein samples were separated by 12% SDS polyacrylamide gel. An electrophoresed gel was stained with InstantBlue protein stain (Expedeon). The purity of protein bands was analyzed by measurement of band intensity in each lane using ImageJ program^35^. The purity of the purified proteins was 94–99%.

### Western blotting analysis

The proteins in SDS-PAGE gel were transferred onto nitrocellulose membrane (Bio-rad). The membrane was incubated with a rabbit polyclonal IgG to human EPO, AB-286-NA (R&D Systems) followed by a donkey derived ECL anti-rabbit IgG, peroxidase-linked species-specific whole antibody, NA934V (GE Healthcare). The signal was developed using an ECL Prime Western Blotting Detection Reagent (GE Healthcare). The chemiluminescent signal was detected using an ImageQuant LAS 4000 machine and analyzed using ImageQuant TL (GE Healthcare).

### Isoform distribution analysis by IEF

BRP and purified EPO samples were buffer exchanged into MilliQ water and separated by an IEF gel with a pH gradient of 2–6 using a flat-bed electrophoresis cell-MultiphorII (GE Healthcare). The electrophoresed samples were then transferred to Armersham Hybond–P PVDF transfer membrane (GE Healthcare) using a semi-dry Novablot transferring system (GE Healthcare). After blotting, the membrane was processed as previously described in Western blot analysis.

### Statistical analysis

*t*-*test* was used to determine the statistical significance differences in the relative T cell response between HLA-DRB1*09-negative and positive groups. One-way ANOVA followed by Tukey’s multiple comparisons test was used to compare the IFN-γ level among test proteins including BRP, EPO-WT, EPO mutants and a positive control, anti-CD3/anti-CD18 antibodies. Analyses were done using GraphPad Prism version 8.4.3 for Windows (Graphpad Software, Inc.).

## Supplementary Information

Supplementary Information.
