# Supplementary material for: Modified recombinant human erythropoietin with potentially reduced immunogenicity
Source: Sci Rep. 2021 Jan 15;11:1491. doi: 10.1038/s41598-020-80402-1 (PMC7810742; doi:10.1038/s41598-020-80402-1)
Supplement: Supplementary file 1 — Supplementary Information. [file 41598_2020_80402_MOESM1_ESM.docx]

Supplementary Information

Title

Modified recombinant human erythropoietin with potentially reduced immunogenicity

Authors

Thanutsorn Susantad^1,2^, Mayuree Fuangthong^2^, Kannan Tharakaraman^3^, Phanthakarn Tit-oon^2^, Mathuros Ruchirawat^2,*^, and Ram Sasisekharan^3,4,*^

Affiliations

^1^Program in Environmental Toxicology, Chulabhorn Graduate Institute, Bangkok, 10210, Thailand

^2^Translational Research Unit, Chulabhorn Research Institute, Bangkok, 10210, Thailand

^3^Koch Institute for Integrative Cancer Research, Massachusetts Institute of Technology, Cambridge, MA 02139, USA

^4^Department of Biological Engineering, Massachusetts Institute of Technology, Cambridge, MA 02139, USA

^*^corresponding author

Contact Information

Email: rams@mit.edu, mathuros@cri.or.th

**Supplementary Table S1.** Determination of HLA-DRB1 types in all volunteers. Blood was collected from healthy volunteers in EDTA‐treated vacuum tubes. High resolution HLA-DRB1 typing was determined using sequence-specific oligonucleotide primed PCR (PCR-SSO) with Luminex-based method at Faculty of Medicine, Chulalongkorn University, Thailand.

| **HLA-DRB1*09 negative volunteers** | **HLA-DRB1*09 positive volunteers** |
| --- | --- |
| Volunteer-negative 1  DRB1*04:05  DRB1*12:01 | Volunteer-positive 1  DRB1*03:01  DRB1*09:01 |
| Volunteer-negative 2  DRB1*07:01/05  DRB1*12:02 | Volunteer-positive 2  DRB1*09:01  DRB1*12:02 |
| Volunteer-negative 3  DRB1*13:02  DRB1*14:01 | Volunteer-positive 3  DRB1*09:01/04  DRB1*16:02 |


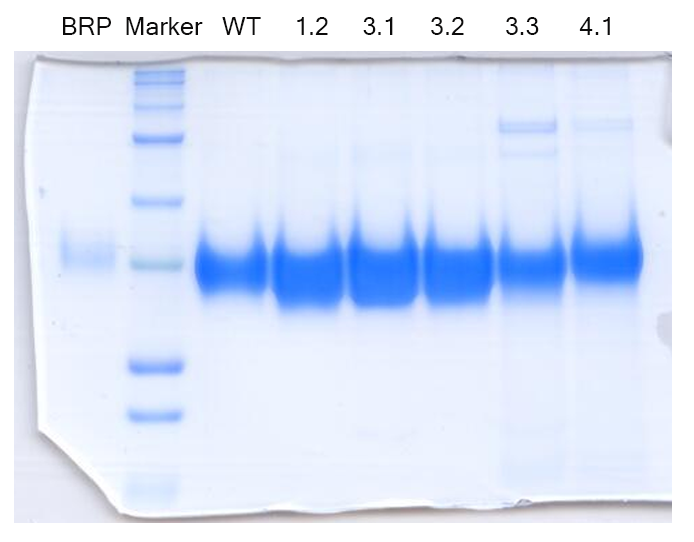


**Supplementary Figure S1. Related to Figure 2a.** Native image of the Coomassie blue stained gel. EPO standard (BRP), wild type and mutant proteins were separated according to their molecular mass in 12% SDS polyacrylamide gel at a constant 175 voltages for 1.5 hr. followed by Coomassie blue staining to visualize the protein purity. The gel image was taken using HP LaserJet scanner. BRP, EPO standard; Marker, Precision Plus Protein Kaleidoscope Prestained Protein Standards; WT, EPO-WT; 1.2, EPO-1.2 (L70V, V74L, L102I); 3.1, EPO-3.1 (T106A); 3.2, EPO-3.2 (T106G); 3.3, EPO-3.3 (T106H); 4.1, EPO-4.1 (L109A).


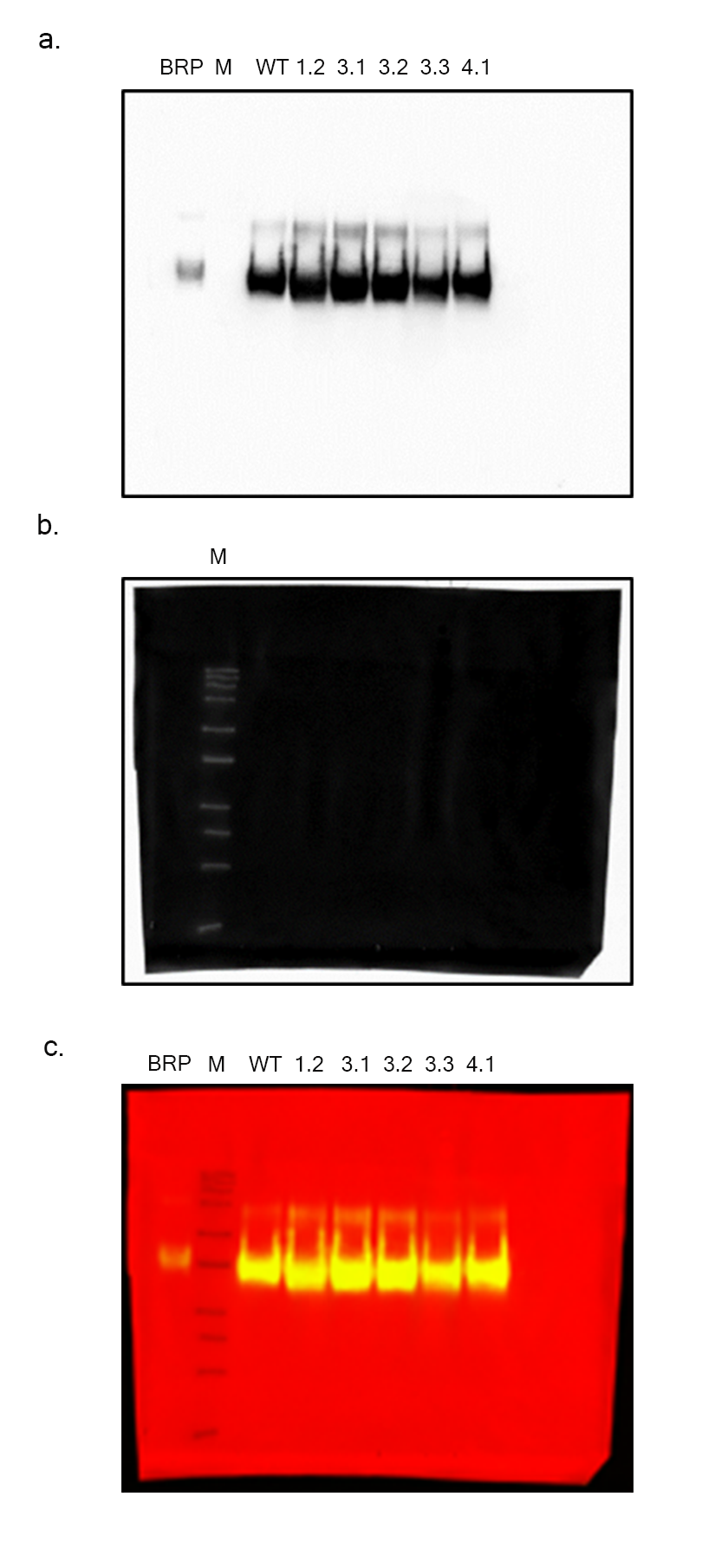


**Supplementary Figure S2. Related to Figure 2b.** Native images of the Western immunoblotting. EPO standard (BRP), wild type and mutant proteins were separated according to their molecular mass in 12% SDS polyacrylamide gel at a constant 175 voltages for 1.5 hr. followed by Western immunoblotting to visualize the presentation of EPO antigen. The chemiluminescence signals of protein samples were detected and the image was taken using ImageQuant LAS 4000 control software version 1.2 provided with ImageQuant LAS 4000 machine at the exposure time of 1 second (a). The signals were then overlaid with the marker (b) using ImageQuant TL software version 7.0 (https://www.cytivalifesciences.com/en/us). The colour display of the overlaid image (c) could not be changed due to the software setting. BRP, EPO standard; M, Precision Plus Protein Kaleidoscope Prestained Protein Standards; WT, EPO-WT; 1.2, EPO-1.2 (L70V, V74L, L102I); 3.1, EPO-3.1 (T106A); 3.2, EPO-3.2 (T106G); 3.3, EPO-3.3 (T106H); 4.1, EPO-4.1 (L109A).

**
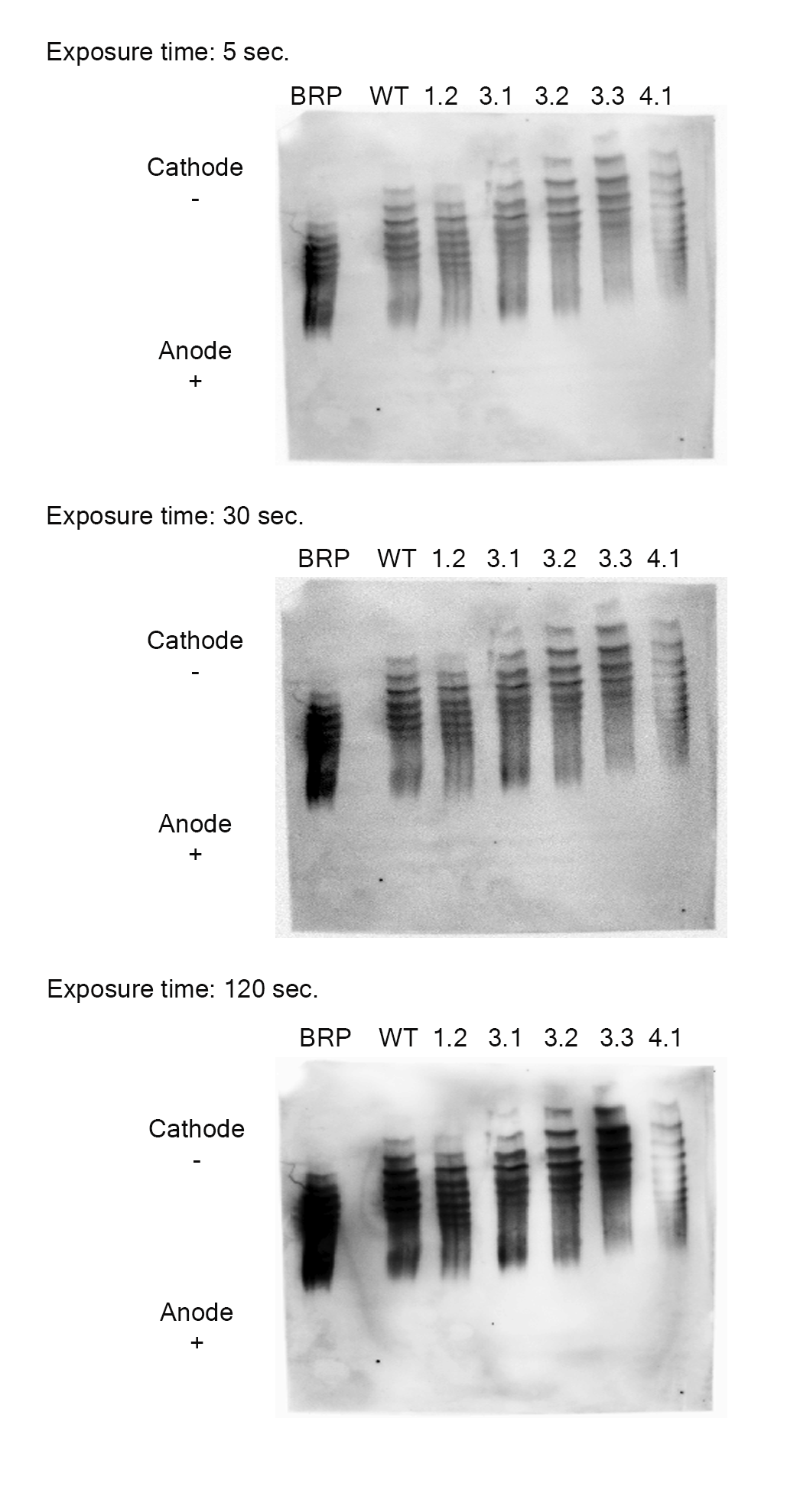
**

**Supplementary Figure S3. Related to Figure 3.** Native images of the isoform analysis using IEF followed by Western immunoblotting with multiple exposures. EPO standard (BRP), wild type and mutant proteins were separated according to their pI in IEF gel with a pH gradient of 2-6 followed by Western blot. The chemiluminescence signals of protein samples were detected and the image was taken using ImageQuant LAS 4000 control software provided with ImageQuant LAS 4000 machine (https://www.cytivalifesciences.com/en/us) at the exposure time of 5 seconds (a), 30 seconds (b) and 120 seconds (c). The separated bands of the proteins represent the number of their existing glycoforms. Distribution of different isoforms of the purified EPO protein was compared to BRP, an EPO standard. BRP, EPO standard; WT, EPO-WT; 1.2, EPO-1.2 (L70V, V74L, L102I); 3.1, EPO-3.1 (T106A); 3.2, EPO-3.2 (T106G); 3.3, EPO-3.3 (T106H); 4.1, EPO-4.1 (L109A).
